# Breaking Up (Amyloid) Is Hard to Do

**DOI:** 10.1371/journal.pmed.0020417

**Published:** 2005-12-27

**Authors:** Sam Gandy, Frank L Heppner

## Abstract

Gandy and Heppner discuss the implications of a new animal study on our understanding of the pathogenesis and treatment of Alzheimer disease.

Standard “Alzheimerology” lore holds that the insolubility of amyloid plaques and neurofibrillary tangles was a great impediment to elucidating the molecular composition of each of these structures. Roughly 77 years passed between Alois Alzheimer's description of the clinical and pathological features of the illness suffered by Auguste D. and the now classical reports from George Glenner, Colin Masters, and Konrad Beyreuther describing partial solubilization, Edman degradation, and primary amino acid sequencing of the Aβ peptide (the protein that accumulates into amyloid plaques) [1–3]. Peter Davies (who discovered the cholinergic deficiency in Alzheimer disease during that same interval [4]) has long joked that one easy way to purify plaques and tangles is to allow the brain from an affected person to autolyze and liquefy completely (“on a summer sidewalk,” according to one colorful variation), whereupon the macro structure of the organ would disintegrate, leaving behind only fibrous clumps and twists.

There is little wonder, then, that the strategy of treating Alzheimer disease with “plaque busting” drugs was relatively slowly embraced: 20 years passed between the availability of amyloid aggregation assays and the clinical trials of the first specific antiamyloid-aggregation compounds. (“Aggregation” is essentially equivalent to clumping, and amyloid clumping can be monitored in a test tube since floating clumps cause light to disperse in a measurable fashion). Conventional wisdom was nihilistic, holding that therapeutic dissolution of amyloid deposits was probably too slow to be approachable. People love to see a dogma challenged, and the availability of mouse models of Alzheimer disease enabled Brad Hyman's group to show, in 2001, that deposits of human Aβ in the transgenic mouse brain were surprisingly dynamic, forming and, unexpectedly, dissolving over a timescale of days [5]. These data injected new optimism into the pursuit of antiamyloid strategies, and by 2005, over 30 discrete compounds or combinations were in development [6].

## Old Worries Resurface

Now, with publication of a study by David Borchelt's group in this issue of *PLoS Medicine* [7], old worries about the efficiency of plaque clearance resurface. David Borchelt, Joanna Jankowsky, and their colleagues report the development of Aβ plaque-forming transgenic mice in which pathology is driven by brain-specific overexpression of a mutated form of the human amyloid precursor protein (APP). The innovation here is that human APP expression due to a genetic trick is extinguishable by adding the antibiotic tetracycline to the mouse food (“tet-off” APP mice). As rightly contended by these authors, the ability to abolish human APP gene expression—instantly and completely—can be conceptually envisioned as equivalent to the most effective antiamyloid strategy imaginable: in other words, a best-case scenario from the point of view of drug efficacy.

**Figure 1:**
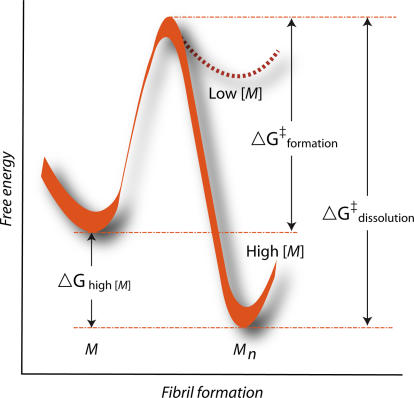
A Large Energy Barrier Prevents Rapid Redissolution of Fibrillar Amyloid In this issue of *PLoS Medicine*, Borchelt and colleagues demonstrate in the living amyloid-laden mouse brain that Aβ plaques are cleared very slowly, even if synthesis of new Aβ precursor molecules is extinguished using a tet-off system [7]. In the recent, relevant, but independent, X-ray crystallography study [8], Nelson et al. envisioned the free-energy plot shown above as a graphic description of the kinetics of transition from monomeric Aβ to fibrillar Aβ (ΔG_formation_). For the reverse reaction, Nelson et al. envision the ΔG_dissolution_ as the large free-energy barrier to spontaneous solubilization of amyloid fibrils. Presumably, it is this ΔG_dissolution_ that underlies the slow disappearance of brain plaques in the Borchelt study in transgenic mice. (Illustration: Sapna Khandwala, adapted from [8])

The rationale was to see how long human amyloid deposits would persist in a plaque-laden mouse brain once new accretion ceased (i.e., once tetracycline switched off new mutated human APP production). The results are arguably applicable to every antiamyloid strategy delivered to patients impaired by Alzheimer disease, since all are believed to enter therapy with at least some existing brain plaque burden. In this tet-off paradigm, unlike the paradigm used earlier by Hyman and colleagues, amyloid pathology was allowed to accumulate, and then human APP expression was completely shut off. Borchelt and colleagues were unable to detect any change in brain amyloid load for at least six months after complete cessation of Aβ biogenesis. The Borchelt data dovetail well with recent biophysical data, proving that the thermodynamic barrier to redissolution of amyloid fibrils is very high indeed (Figure 1) [8].

## Implications for Therapy and Prevention

If this mouse model represents the best-case scenario, what are the realistic hopes of success for anti-Aβ therapies in the treatment of human Alzheimer disease? Several points come to mind. First, the recent proposal that oligomeric/dodecameric forms of Aβ (also known as Aβ-derived diffusible ligands [ADDLs]) are the real culprits in mediating Aβ neurotoxicity [9] provides some hope that ADDLs, not Aβ plaques, represent the most important Aβ biophysical form that must be purged in order to cause a therapeutic benefit. Borchelt and colleagues have not yet determined what happens to ADDL levels in the brains of their tet-off APP mice, but such data are eagerly anticipated.

Second, Borchelt and colleagues' data are consistent with the evidence that all model antiamyloid strategies are superior when initiated long before onset of amyloidosis. Such evidence fuels a new initiative of the United States Alzheimer's Association aimed not at treating but at preventing Alzheimer disease (http://www.alz.org/maintainyourbrain). Interventional strategies early in life should not only prevent amyloid pathology, but also take advantage of the greater regenerative capacity of the human brain at younger ages. The wisdom of preventing Alzheimer disease is further galvanized by the appreciation over the last five years of evidence that many modifiable diet and lifestyle factors (body mass index, cholesterol levels, control of diabetes and blood pressure, and mental and physical exercise) may modulate risk for late-life degenerative dementia [10].

## The Challenges Ahead

Significant challenges lie ahead in understanding how these risks and pathologies are intertwined, but the aging of the baby boom population promises an unprecedented epidemic of dementia if effective interventions are not discovered soon. In the US, the population of individuals age 65 years and over is the fastest growing segment in society, and one-half of individuals over 85 experience dementia [11]. On a practical level, this means that almost everyone will be either a patient or a caregiver in the near future. Disasters such as tsunamis and hurricanes—as painful, dramatic, and expensive as they are—pale in comparison to the cataclysm our aging societies will face if current trends in the epidemiology of this dementing illness continue on unchecked.

## Abbreviations

ADDL: Aβ-derived diffusible ligand
APP: amyloid precursor protein
